# Extracellular vesicles and acetylation: reciprocal regulation in disease progression

**DOI:** 10.3389/fimmu.2026.1890445

**Published:** 2026-08-03

**Authors:** Yang Liu, Sinan Wu, Yaqian Wang, Xiali Zhu, Le Kang

**Affiliations:** School of Pharmacy, Henan University of Chinese Medicine, Zhengzhou, China

**Keywords:** acetylation, exosomes, extracellular vesicles, histone acetylation, histone deacetylase inhibitors

## Abstract

Extracellular vesicles (EVs) are membrane-bound particles secreted by diverse cell types and mediate intercellular communication through the transfer of proteins, RNAs, lipids, metabolites and other biomolecules. EVs participate in multiple physiological and pathological processes, including immune regulation, tumor progression, metastasis, neurodegenerative disorders and cardiovascular disease. Acetylation is a dynamic post-translational and epigenetic modification that regulates protein function, chromatin accessibility, transcription, metabolism and immune responses. Emerging evidence indicates a bidirectional regulatory relationship between EVs and acetylation. Acetylation can regulate EV biogenesis, cargo loading, secretion and EV-associated protein trafficking, whereas EVs can reshape intracellular acetylation states in recipient cells by transferring non-coding RNAs, proteins, metabolites and acetylated proteins. In this review, we summarize the mechanisms by which acetylation controls EV generation and cargo composition, and how EVs, in turn, modulate acetyl-CoA metabolism, acetylation writers and erasers, and acetylated protein signaling in recipient cells. Given the relevance of this axis to cancer immunity, we also discuss its implications for antigen presentation, macrophage polarization, CD8^+^ T-cell dysfunction, regulatory T cells, natural killer cells and immune checkpoint regulation. Furthermore, we evaluate the biomarker potential of EV-associated acetylation signatures, the therapeutic relevance of histone deacetylase inhibitors and EV-based interventions, and the current methodological and translational limitations of the field. This review provides a conceptual framework for understanding the EV-acetylation axis in disease progression and highlights future directions for immunological, biomarker and therapeutic development.

## 1 Introduction

Extracellular vesicles (EVs) are membrane-enclosed nanoparticles secreted by virtually all cell types and mediate intercellular communication through the transfer of proteins, nucleic acids, lipids, and metabolites (1–3). In disease, tumor-derived EVs remodel the microenvironment by delivering bioactive cargo to immune cells, fibroblasts, and stromal populations, thereby promoting tumor growth, metastasis, and therapeutic resistance (4–6). EVs secreted by non-malignant stromal cells can also reciprocally influence tumor cell behavior (7–9). These properties establish EVs as mediators of pathological signaling and potential therapeutic targets.

Acetylation is a key post-translational modification that primarily occurs on lysine residues and is catalyzed by acetyltransferases (10–12). By regulating protein stability, activity, molecular interactions, gene expression, chromatin structure, and cell signaling, acetylation participates in multiple cellular processes (13–15). Dysregulated acetylation is associated with cancer, neurodegenerative diseases, and cardiovascular disorders (16–18). In cancer, abnormal acetylation may contribute to tumor suppressor inactivation, oncogene activation, metabolic remodeling, and immune regulation (19–21). In neurodegenerative diseases such as Alzheimer’s disease, altered acetylation may disrupt neuronal function and survival (22, 23). Therefore, acetylation-related mechanisms may provide potential therapeutic targets.

A recent study showed that acetylation modifications can affect disease progression by influencing EV generation and cargo composition (24). Conversely, EVs can modulate intracellular acetylation patterns in specific pathological contexts (25). However, these observations have largely been reported in isolated models, and a systematic integration of the bidirectional EV–acetylation axis across disease settings is still lacking. In this review, we summarize how acetylation controls EV biogenesis and cargo loading, how EVs reshape intracellular acetylation through acetyl-CoA metabolism, acetylation writers and erasers, and acetylated protein transport, and how this axis may be targeted for disease intervention.

## 2 Biogenesis and functional diversity of extracellular vesicles

**Figure 1:**
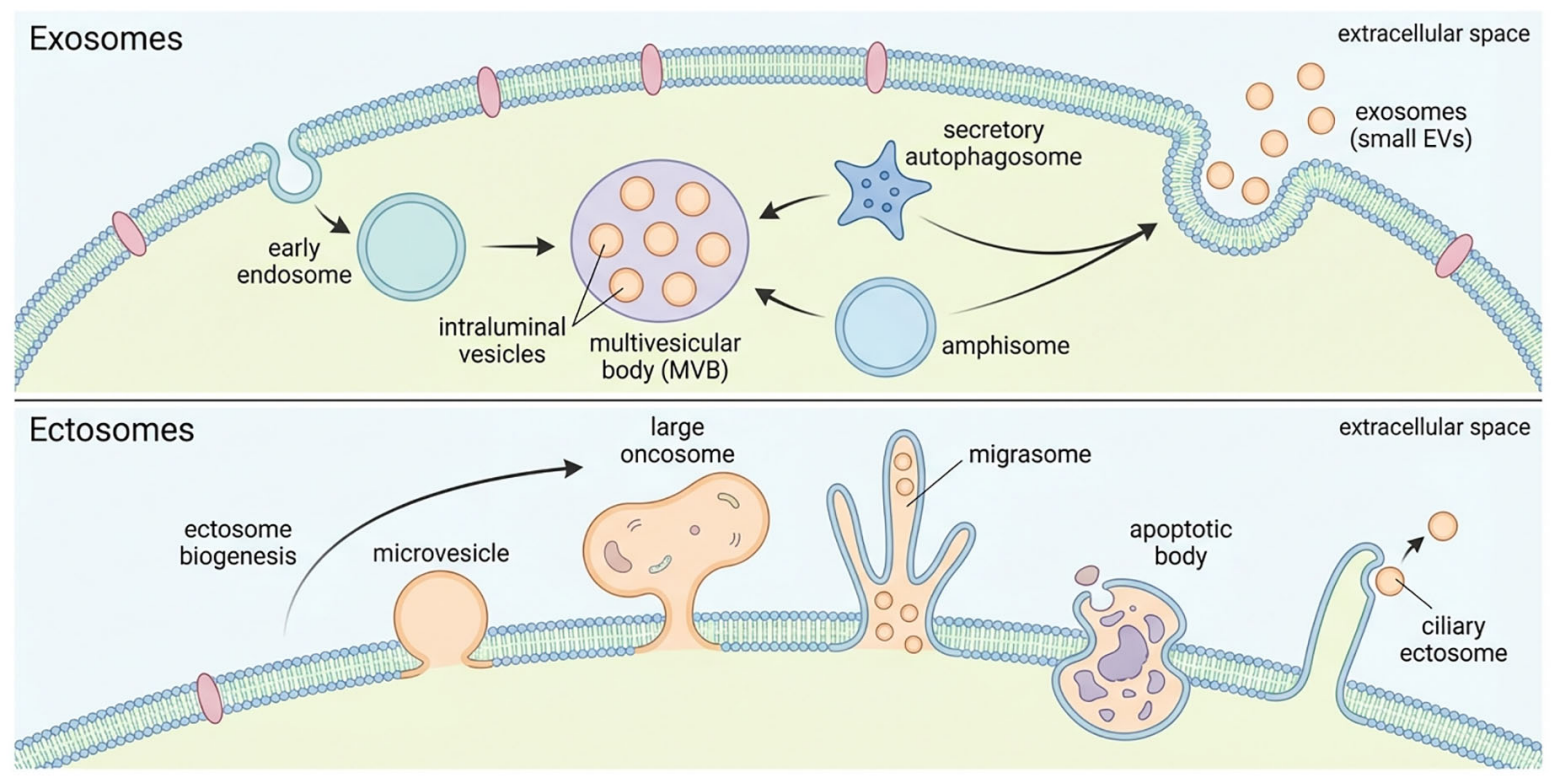
Biogenesis pathways and diversity of extracellular vesicles (EVs). Historically, EVs have often been broadly classified into two main types based on their presumed biogenesis: exosomes and microvesicles. Exosomes are small EVs derived from the endosomal system and are released when multivesicular bodies (MVBs) or amphisomes fuse with the plasma membrane. Amphisomes arise from the fusion of autophagosomes with MVBs. In contrast, microvesicles are generated by outward budding and fission of the plasma membrane. EV populations are heterogeneous and may also include larger vesicles such as oncosomes and apoptotic bodies. Tumor cells, immune cells, and other stromal cells can release distinct EV subtypes that differ in size, cargo composition and function, thereby contributing to intercellular communication in both physiological and pathological conditions.

EV-related membrane vesicles and vesicle-like structures had been described before the umbrella terminology became standardized. In this review, we use the term “extracellular vesicles” to refer broadly to cell-derived particles enclosed by a lipid bilayer, consistent with contemporary EV nomenclature and recommendations (26). EVs are released by virtually all cell types, including tumor cells, fibroblasts, macrophages, endothelial cells, and other stromal or immune cell populations. Historically, EVs have often been classified according to their presumed biogenesis into endosome-derived exosomes and plasma membrane-derived ectosomes or microvesicles (27–29). However, the current MISEV recommendations caution against assigning EV subtypes solely on the basis of size, density, or common marker expression unless their subcellular origin has been unequivocally demonstrated (30). Accordingly, small EVs should be regarded as an operational size-based category rather than as synonymous with exosomes, because small EV preparations may contain vesicles generated through different biogenetic routes, including plasma membrane-derived vesicles. Therefore, throughout this review, we use the term EVs as a general descriptor and specify exosomes, microvesicles, or other subtypes only when the original studies provide sufficient evidence for their biogenesis or when these terms are used in the cited literature. Operationally, EVs can be described according to physical characteristics such as size and density, biochemical composition such as surface or intravesicular markers, and biological source such as donor cell type or body fluid. In this framework, small EVs are commonly defined as particles below approximately 200 nm, whereas medium or large EVs are generally larger than 200 nm. The apparent abundance of different EV subpopulations varies substantially depending on donor cell type, biological fluid, physiological or pathological state, pre-analytical variables, and isolation methodology. Therefore, the relative abundance of small and large EVs should not be generalized across all experimental systems. The major biogenesis routes and updated classification of EV subpopulations are summarized below (**Figure 1**; **Table 1**).

**Table 1:** Operational and historical classification of extracellular vesicle subpopulations.

EV category	Approximate size	Main defining features
Small EVs	*<*200 nm	Operational size-based group; commonly enriched in CD9, CD63, CD81, TSG101, or ALIX
Large EVs	*>*200 nm	Operational size-based group with heterogeneous origins and cargo composition
Exosomes	30–150 nm	Endosome-derived EVs released after multivesicular body fusion with the plasma membrane
Microvesicles or ectosomes	100–1000 nm	Plasma membrane-derived EVs generated by outward budding and fission
Apoptotic bodies	100–5000 nm	Vesicles generated during apoptotic cell disassembly; may contain nuclear or organelle fragments
Migrasomes	0.3–5 *µ*m	Vesicles formed on retraction fibers during cell migration
Large oncosomes	5–20 *µ*m	Large tumor-derived EVs associated with oncogenic stress, membrane blebbing, and cancer progression
Blebbisomes	≤20*µ*m	Large organelle-rich EVs with active blebbing behavior, cell-like properties, and immunoregulatory cargo

A recently described large EV subtype, termed blebbisomes, further illustrates the expanding heterogeneity of EV biology. Jeppesen et al. reported that cells can actively release exceptionally large membrane-enclosed vesicles up to approximately 20 *µ*m in diameter, which contain cellular organelles such as functional mitochondria and multivesicular endosomes, lack a definable nucleus, and exhibit active blebbing behavior (31). Notably, cancer-derived blebbisomes were shown to contain multiple inhibitory immune checkpoint proteins, including PD-L1, PD-L2, B7-H3, VISTA, PVR, and HLA-E, suggesting potential relevance to tumor immune evasion. A recent review further highlighted that large EVs and blebbisomes may contribute to tumor progression, immune modulation, metabolic reprogramming, premetastatic niche formation, and translational applications in cancer diagnosis and therapy (32). However, the biogenesis, molecular markers, purification strategies, *in vivo* behavior, and functional specificity of blebbisomes remain incompletely defined. Although blebbisomes have not yet been directly linked to acetylation-dependent regulation, their capacity to carry bulky cargos, organelles, and immunoregulatory molecules raises the possibility that they may participate in the transfer of acetylation-related enzymes, metabolic organelles, or acetylated proteins in specific disease contexts.

Previous research has often focused on vesicles described as exosomes in the original literature or on exosome-enriched small EV preparations. Such studies have contributed to the current understanding of endosomal EV biogenesis, although the precise subcellular origin of EV preparations should be interpreted according to the characterization methods used in each study. The assembly of these vesicles involves the coordinated action of several key proteins. Initially, the ESCRT complexes, including ESCRT-0, ESCRT-I, ESCRT-II, and ESCRT-III, participate in the formation of multivesicular bodies by promoting endosomal membrane invagination and budding (33, 34). ESCRT-III components, such as CHMP proteins and the Vps4 disassembling factor, are important for vesicle scission (35). The Rab GTPase family, especially Rab27a and Rab27b, regulates the transport and extracellular release of EVs (36). In addition, lipids such as ceramide contribute to vesicle formation by enhancing membrane curvature (37), while membrane-associated proteins such as flotillin are involved in EV biogenesis and trafficking (38). Proteins such as ALIX and syntenin also act as cofactors during EV assembly and may assist ESCRT-associated processes (39). The coordination of these molecular pathways enables EVs to participate in intercellular communication and signal transfer.

Because EV preparations are heterogeneous, rigorous characterization is essential. Rather than relying on a single marker or size range to define an EV subtype, multiple complementary approaches should be used. Physical properties can be assessed by nanoparticle tracking analysis (NTA), transmission electron microscopy (TEM), or related imaging and sizing methods. Biochemical composition can be evaluated by detecting EV-associated markers such as CD9, CD63, CD81, TSG101, or ALIX, together with negative markers that indicate contamination from intracellular organelles, such as calnexin (40, 41). These criteria are particularly important when studies aim to distinguish small EVs, exosome-like vesicles, microvesicles, or other EV subpopulations.

EVs contain diverse biomolecules, including proteins, lipids, RNA species such as miRNAs, lncRNAs, and circRNAs, DNA, and metabolites (42, 43). These cargos allow EVs to regulate the behavior of recipient cells by influencing signaling pathways, gene expression, metabolism, immune responses, and cell migration. In the context of this review, particular attention should be paid to EV cargos that intersect with acetylation biology, including non-coding RNAs that regulate acetylation-related enzymes, proteins that influence acetyl-CoA metabolism, acetylation writers and erasers, and acetylated proteins themselves. These cargo categories provide the mechanistic basis for the reciprocal EV-acetylation axis discussed below.

**Figure 2:**
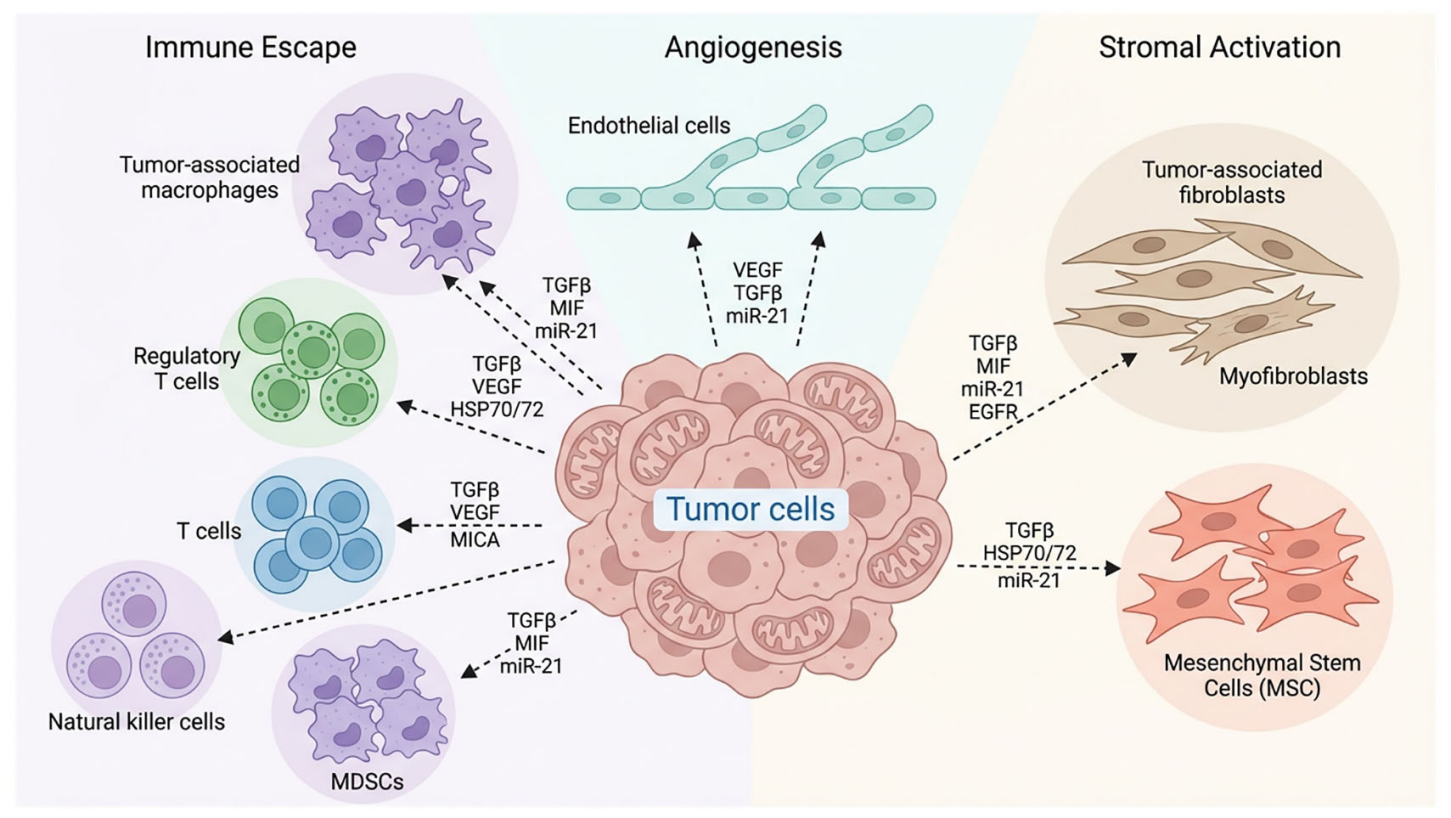
Role of extracellular vesicles (EVs) in tumor microenvironment communication. Tumor cells, immune cells, fibroblasts, endothelial cells and other stromal populations release heterogeneous EVs that transfer proteins, nucleic acids, lipids and metabolites to recipient cells. In the tumor microenvironment, EV-mediated communication can promote tumor growth, angiogenesis, extracellular matrix remodeling, cancer-associated fibroblast activation, macrophage polarization, immune suppression, metastatic niche formation and therapeutic resistance. Conversely, EVs derived from immune or stromal cells may either support or restrain antitumor responses depending on their cellular source, activation state and cargo composition.

Recent studies on the role of EVs in disease initiation and progression have provided evidence that EV production can actively promote pathological processes. In cancer, for example, tumor-derived EVs can alter the behavior of immune and stromal cells in the tumor microenvironment, thereby fostering pro-cancer activities such as macrophage polarization toward an M2-like phenotype (44, 45) (**Figure 2**). In addition, EVs released by non-tumor cells within the tumor microenvironment can contribute to cancer progression, therapy resistance, and immune escape (46, 47). Beyond cancer, EVs also participate in cardiovascular disorders and diseases of the digestive system (48, 49). Consequently, targeting the production, release, uptake, or cargo function of EVs in disease states has emerged as a promising therapeutic approach, particularly for complex diseases involving multiple interacting cell types.

## 3 Acetylation modification in physiology and disease

**Figure 3:**
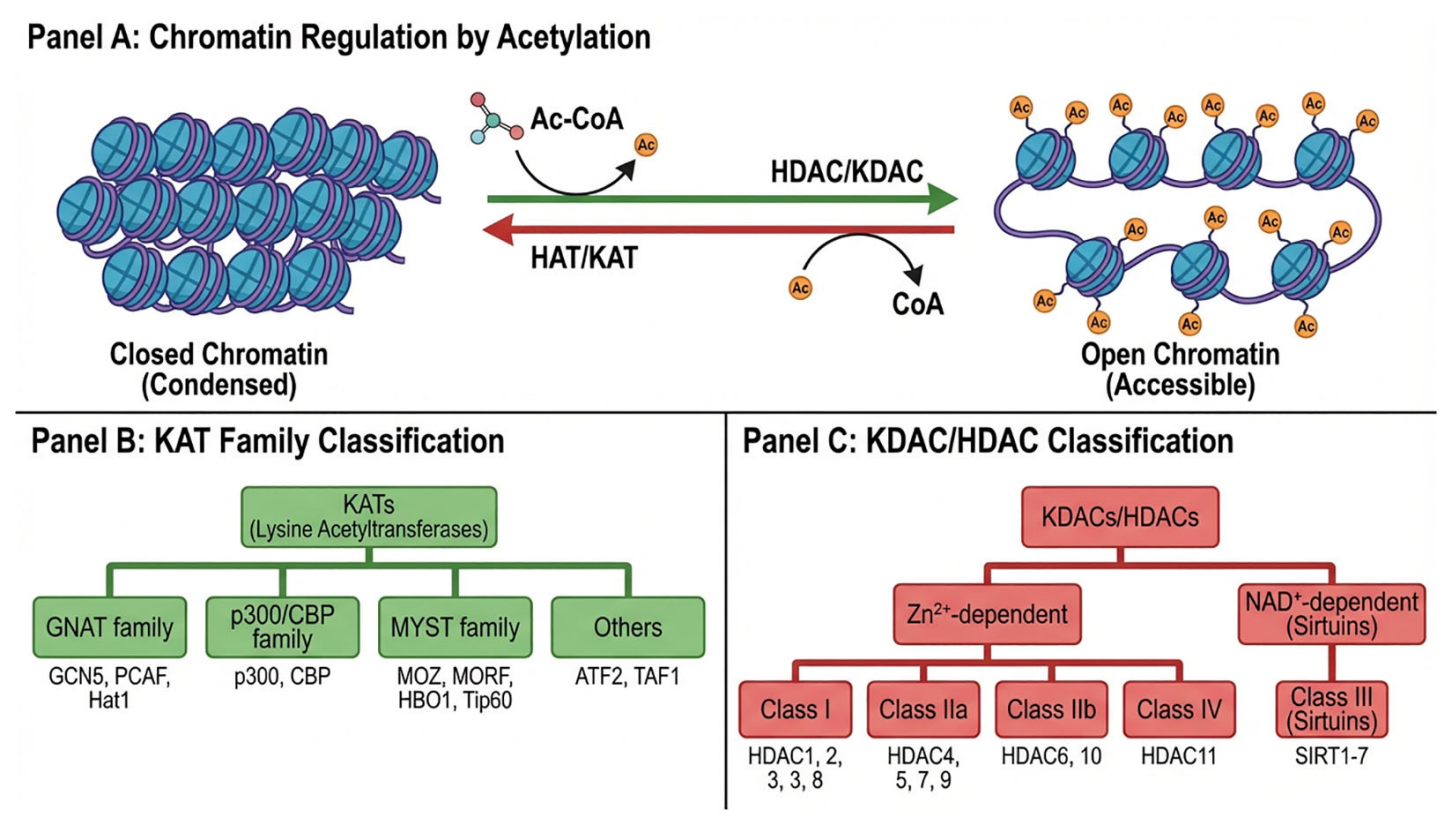
Regulation of the reversible lysine acetylation process. **(A)** Schematic representation of reversible histone acetylation. Histone acetyltransferases (HATs) transfer acetyl groups (Ac) from coenzyme A (CoA) to lysine residues on histone tails, leading to chromatin relaxation and transcriptional activation, whereas histone deacetylases (HDACs) remove acetyl groups and promote chromatin compaction. **(B)** Major classes of lysine acetyltransferases (KATs/HATs), including GNAT (e.g. GCN5, PCAF), p300/CBP and the MYST family (e.g. Tip60, MOZ, MORF, HBO1, MOF). **(C)** Major classes of lysine deacetylases (KDACs/HDACs): Zn^2+^-dependent “classical” HDACs (classes I, IIa, IIb and IV) and NAD^+^-dependent sirtuins (class III). CBP, CREB-binding protein; GCN5, general control of amino acid synthesis protein 5; GNAT, GCN5-related N-acetyltransferase; HAT, histone acetyltransferase; HBO1, histone acetyltransferase binding to ORC1; HDAC, histone deacetylase; KAT, lysine acetyltransferase; KDAC, lysine deacetylase; MOF, males absent on the first; MORF, monocyte leukemia zinc finger-related factor; MOZ, monocytic leukemia zinc finger protein; MYST, MOZ/Ybf2 (Sas3)/Sas2/Tip60; NAD, nicotinamide adenine dinucleotide; PCAF, p300/CBP-associated factor; RNAPII, RNA polymerase II; SIRT, sirtuin deacetylase; Tip60, 60kDa Tat-interacting protein.

Acetylation is a fundamental post-translational modification in which an acetyl group (CH_3_CO) is covalently attached to specific amino acid residues, most commonly lysine, via the catalytic activity of acetyltransferases (50–52) (**Figure 3A**). This modification can reshape protein conformation, modulate protein–protein and protein–DNA interactions, and thereby alter protein activity and stability (53, 54). Through these effects, acetylation exerts broad influence on cellular functions. Beyond its well-established role in gene expression regulation, acetylation participates in a variety of biological processes, including cell cycle control, metabolic pathway regulation, and the modulation of innate and adaptive immune responses (55–57). Its impact extends from chromatin architecture and transcription factor function to signal transduction and cellular adaptation to stress (19). With the deepening of research, acetylation is increasingly recognized as a finely tuned molecular “switch” that dynamically responds to environmental and physiological cues.

The acetylation status of proteins is determined primarily by two opposing enzyme families: histone acetyltransferases (HATs) and histone deacetylases (HDACs) (**Figures 3B, C**). HATs (also referred to as lysine acetyltransferases, KATs) transfer an acetyl group from acetyl-CoA to the *ϵ*-amino group of lysine residues on target proteins, which are often located at the N-terminal tails or other functionally critical regions (58–60). This modification can disrupt electrostatic interactions, alter higher-order protein structure, and promote or inhibit the binding of interacting partners, thereby reshaping protein function. In contrast, histone deacetylases (also termed lysine deacetylases, KDACs) remove acetyl groups from lysine residues, restoring the positively charged state of the side chain and reversing the functional consequences of acetylation (61–63). The dynamic equilibrium between HAT/KAT and HDAC/KDAC activities is crucial for maintaining cellular homeostasis. By precisely modulating the activities of these enzymes, cells achieve temporal and spatial control of acetylation, which in turn regulates key processes such as gene transcription, DNA damage repair, and cell cycle progression (64, 65). Consequently, dysregulation of HATs and HDACs is closely linked to the onset and progression of multiple diseases, including various cancers, neurodegenerative disorders, and metabolic syndromes.

Acetylation plays a particularly critical role in histones and chromatin regulation. Histones are core components of chromatin, and lysine residues in their N-terminal tails are prominent substrates for acetylation (66, 67). Acetylation of these lysines neutralizes their positive charge, weakens the interaction between histones and DNA, and leads to chromatin relaxation. This relaxed chromatin state increases the accessibility of DNA to transcription factors and other regulatory complexes, thereby facilitating transcription initiation and elongation (68). As a key epigenetic mechanism, histone acetylation enables cells to flexibly regulate gene expression in response to extracellular stimuli and intracellular metabolic signals, supporting rapid adaptation to environmental changes.

In addition to histones, acetylation is widely distributed on non-histone proteins and profoundly influences their function and stability (69). A broad spectrum of non-histone substrates, including enzymes, receptors, transcription factors, and signaling molecules, is subject to dynamic acetylation–deacetylation cycles (70). For instance, acetylation can modulate the stability, subcellular localization, and DNA-binding capacity of transcription factors, thereby reprogramming downstream signaling pathways. In some cases, acetylation enhances enzyme catalytic activity or alters substrate specificity; in others, it participates in the regulation of the cell cycle, apoptosis, and multiple metabolic pathways. Collectively, these findings highlight that lysine acetylation is not restricted to chromatin-level regulation but acts as a global regulatory mechanism that integrates transcriptional control with cellular signaling and metabolism.

**Figure 4:**
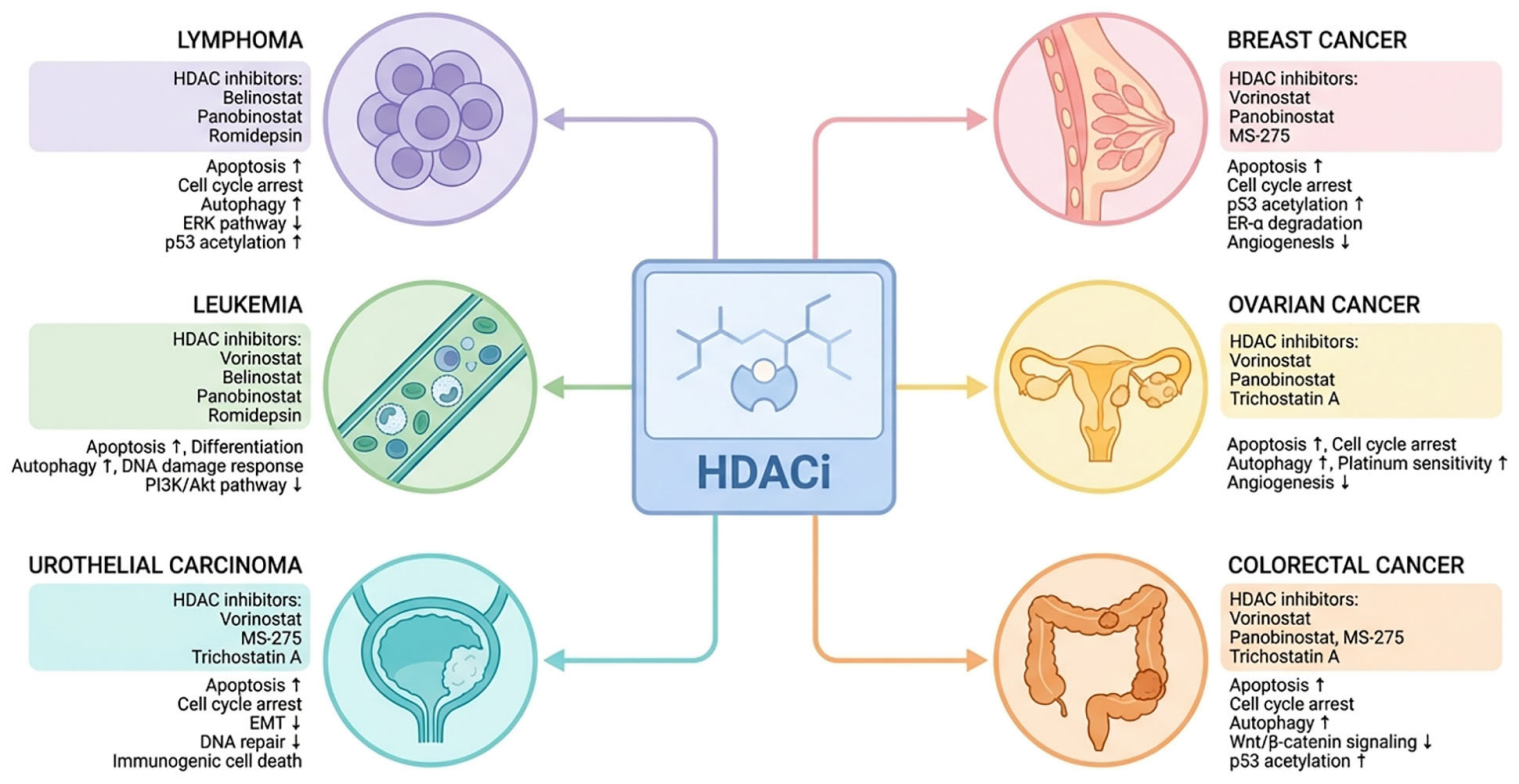
Schematic overview of histone deacetylase inhibitors (HDACi) used in tumor progression treatment. HDACi increase acetylation of histone and non-histone proteins, thereby modulating gene expression, inducing cell cycle arrest and apoptosis, and enhancing sensitivity to radiotherapy and chemotherapy in cancer cells.

In summary, acetylation is an indispensable post-translational modification that orchestrates gene regulation, maintains cellular function, and participates in the pathogenesis of numerous diseases. As a dynamic and reversible regulatory mechanism, acetylation senses and responds to changes in both intracellular and extracellular environments, thereby shaping cellular fate and biological behavior. Theoretically, both histone and non-histone proteins can undergo acetylation. In disease contexts, acetylation may act on molecular targets that either promote or suppress disease progression, indicating that global acetylation levels alone do not directly determine disease outcomes. Nonetheless, accumulating evidence suggests that aberrant acetylation patterns are closely involved in disease development. For example, histone deacetylase inhibitors (HDACi) have been shown to suppress tumor growth and progression in various models (**Figure 4**) (71, 72). Moreover, HDACi have demonstrated therapeutic potential beyond oncology, attenuating disease progression in movement disorders, cardiovascular diseases, gastrointestinal disorders, metabolic syndromes, and respiratory diseases (73–76). These observations indicate that modulation of cellular acetylation status, either by enhancing acetylation or by inhibiting deacetylation, can exert beneficial effects on multiple pathological processes. Consequently, targeting acetylation-related enzymes, particularly HDACs, has emerged as a promising strategy for the prevention and treatment of a broad spectrum of human diseases. Notably, several of these beneficial effects of HDACi are increasingly being linked to EV-mediated intercellular communication, suggesting that acetylation and EV biology are mechanistically intertwined.

## 4 Reciprocal regulation between acetylation and extracellular vesicles

**Figure 5:**
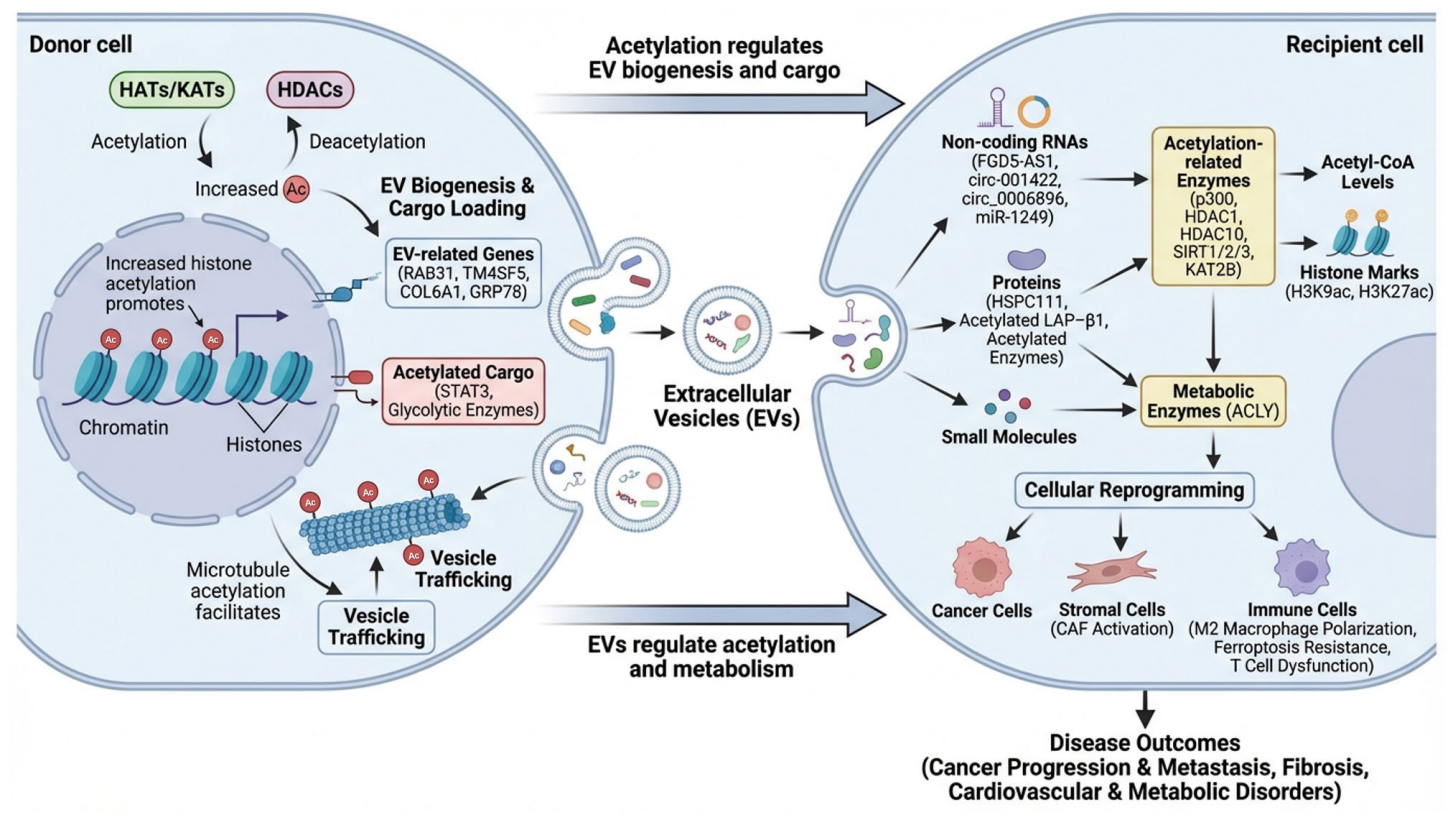
Schematic model of the bidirectional regulatory axis between extracellular vesicles (EVs) and acetylation in disease progression. On the donor side, histone and non-histone acetylation, controlled by histone acetyltransferases (HATs/KATs) and histone deacetylases (HDACs), regulates the transcription of EV-related genes (e.g. *RAB31*, *COL6A1*, *GRP78*) and the acetylation status of cargo proteins such as STAT3 and glycolytic enzymes. Microtubule acetylation and the TM4SF5–HDAC6–SLAC2B complex further promote vesicle trafficking and EV release via multivesicular bodies and plasma membrane budding. The resulting EVs carry diverse cargos, including non-coding RNAs (FGD5-AS1, circ-001422, circ 0006896, miR-1249), proteins (HSPC111, acetylated LAP–TGF-*β*1, acetylated glycolytic enzymes) and small molecules. On the recipient side, EV uptake by cancer cells, cancer-associated fibroblasts, macrophages, CD8^+^ T cells and endothelial cells delivers these cargos, which modulate acetylation-related enzymes (e.g. p300, HDAC1, HDAC10, SIRT1/2/3, HDAC2/3, KAT2B) and metabolic enzymes such as ATPcitrate lyase (ACLY), thereby altering acetyl-CoA levels and histone marks (e.g. H3K9ac, H3K27ac). These changes reprogram gene expression, drive cancer-associated fibroblast activation, M2 macrophage polarization, ferroptosis resistance and T cell dysfunction, and ultimately contribute to cancer progression and metastasis, fibrosis, and cardiovascular and metabolic disorders. This figure represents a conceptual synthesis of mechanisms reported in different experimental systems and should not be interpreted as a single validated pathway operating uniformly across all disease contexts.

The relationship between acetylation and extracellular vesicles has attracted increasing attention. On the one hand, EVs produced under pathological conditions contribute to disease progression, and HDACi can slow the progression of multiple diseases by reshaping acetylation patterns. On the other hand, emerging evidence suggests that acetylation itself can regulate EV biogenesis, cargo loading and secretion, while EVs, in turn, can remodel intracellular acetylation states in recipient cells (24, 25). These findings support the existence of a bidirectional EV–acetylation axis that may synergistically regulate disease progression (**Figure 5**).

Consistent with this concept, Chen et al. provided compelling evidence that the therapeutic effects of HDACi may, at least in part, depend on EV-mediated communication. They showed that trichostatin A (TSA)–induced hyperacetylation enhances myotube fusion and promotes the secretion of miR-8733p-enriched EVs. These TSA-EVs markedly enhance osteogenic differentiation in human bone marrow mesenchymal stem cells by targeting calponin-2 (CNN2), leading to increased bone mass (24). In another study, Zhang et al. demonstrated that acetylation in osteosarcoma cells upregulates *COL6A1* expression, which is subsequently transferred to fibroblasts via exosomes, driving their conversion into tumor-associated fibroblasts and thereby accelerating osteosarcoma progression (77). These observations suggest that acetylation modifications can critically influence the production and functional output of EVs, ultimately affecting disease progression.

More direct mechanistic evidence was reported by Khanal et al., who showed that enhanced acetylation at histone H3 lysine 9 (H3K9ac) in the promoter region increases the expression of EV-related genes such as Ras-related protein Rab-31 (RAB31), leading to augmented EV release and exacerbation of liver fibrosis (78). In addition, Kim et al. found that the accumulation of transmembrane 4 L six-family member 5 (TM4SF5) in vesicles depends on microtubule acetylation. A trimeric complex composed of TM4SF5, HDAC6, and SLAC2B accumulates in the perinuclear region and subsequently moves toward the leading edge, indicating that cytoskeletal acetylation can regulate the trafficking and vesicular localization of TM4SF5 (79). Similarly, Xu et al. demonstrated that acetylation of STAT3 affects its interaction with Sec15b, a component of the exocyst complex (EXOC6B), thereby modulating the accumulation of STAT3 in EVs (80). Collectively, these studies indicate that acetylation can regulate the synthesis, packaging, and release of EVs either by acting on the promoter regions of EV-related genes or by directly modifying EVassociated proteins. Furthermore, HDACi can modulate the secretion of acetylation-dependent cargo such as GRP78 (81), suggesting that pharmacological manipulation of acetylation may influence EV-associated protein release.

Conversely, EVs can also regulate acetylation modifications in recipient cells. For example, Haque et al. reported that EVs derived from adipose tissue in obesity promote acetylation in recipient cells (82). Ying et al. found that EVs from tumor cells are able to modulate acetylation patterns in endothelial cells (83), although the underlying mechanisms remained unclear in that study. This mechanistic gap was partly addressed by Zhang et al., who showed that exosomes secreted by colorectal cancer cells deliver HSPC111 into cancer-associated fibroblasts. In these fibroblasts, HSPC111 regulates lipid metabolism by promoting the phosphorylation of ATP-citrate lyase (ACLY), thereby increasing acetyl-CoA production. Elevated acetyl-CoA levels enhance H3K27 acetylation, which in turn upregulates *CXCL5* expression and secretion, ultimately promoting colorectal cancer liver metastasis (84). This study provides a clear example of how tumor-derived exosomes can reshape the acetylation landscape of stromal cells by modulating metabolic enzymes and acetyl-CoA availability.

EVs can also regulate acetylation by targeting acetylation-related enzymes, including both writers and erasers. He et al. showed that FGD5-AS1 in pancreatic cancer-derived exosomes interacts with p300 in macrophages, leading to increased STAT3 acetylation. This modification enhances STAT3/NF-*κ*B nuclear localization and transcriptional activity, thereby promoting M2 macrophage polarization and tumor progression (25). Similarly, Cao et al. reported that exosomal circ-001422 binds to p300, resulting in STAT3 acetylation and activation of the STAT3/NF-*κ*B axis, which likewise drives M2 macrophage polarization and accelerates glioma progression (85). In acute myeloid leukemia, Can et al. found that EVs secreted by leukemia cells carry circ 0006896, which interacts with the catalytic domain of HDAC1. This interaction reduces histone H3 acetylation and alters the transcription of genes involved in arachidonic acid metabolism, thereby inhibiting lipid peroxidation and ferroptosis in leukemia cells. Furthermore, exosomal circ 0006896 binds HDAC1 in CD8^+^ T cells, impairs *LEF1* transcription, and decreases the expression of cytotoxic molecules such as IFN-*γ* and granzyme B, ultimately dampening antitumor immunity (86).

In a similar vein, Su et al. demonstrated that miR-1249 contained in endothelial cell-derived EVs regulates cigarette smoke-induced pulmonary hypertension by inhibiting HDAC10 and the NF-*κ*B–CaSR cascade (87). In addition to non-coding RNAs, small molecules and other EV cargo can modulate intracellular acetylation by targeting critical acetylation-related enzymes such as SIRT1, SIRT2, SIRT3, HDAC2, HDAC3 and KAT2B (88–95). Altogether, these findings indicate that EVs regulate intracellular acetylation either by influencing acetyl-CoA metabolism or by directly modulating acetylation-modifying proteins, thereby contributing to disease progression.

Acetylated proteins transported via EVs may also provide an additional layer of EV–acetylation crosstalk in disease progression. Minic et al. reported that breast cancer-derived small EVs contain glycolysis-related enzymes with distinct lysine acetylation patterns and increased enzymatic activity (96). These findings suggest that EV-associated acetylated metabolic enzymes may be delivered to recipient tumor or stromal cells and thereby influence glycolytic flux. Enhanced glycolysis can increase lactate production, and lactate accumulation may create a metabolic environment permissive for histone lactylation, including H3K18 lactylation, which has been implicated in tumor progression, macrophage polarization, immune suppression, and therapy resistance (97–102). Thus, EV-mediated transfer of acetylated glycolytic enzymes may establish a feed-forward model in which acetylation-dependent EV cargo indirectly promotes lactate-driven epigenetic remodeling in recipient cells. However, this acetylation-to-lactylation connection remains hypothetical, and direct evidence showing that EV-delivered acetylated enzymes increase lactate levels and induce histone lactylation is still lacking. Future studies should combine EV acetylome profiling, metabolic flux analysis, lactylome mapping, and functional perturbation of specific acetylated enzyme sites to test this model.

Consistent with the broader relevance of acetylated EV cargo, Yu et al. reported that acetylated LAP–TGF-*β*1 protein, when incorporated into exosomes, promotes the dissemination of triple-negative breast cancer cells in lung micrometastasis (115). These observations suggest that acetylated proteins are not merely passive EV cargos, but may actively participate in intercellular signaling, metabolic remodeling, and disease progression. To facilitate comparison among representative studies, the currently reported EV-acetylation mechanisms are summarized in **Table 2**.

**Table 2:** Representative studies supporting the EV–acetylation axis in disease progression.

EV cargo/regulator	Acetylation link	Cell source/target	Main effect	Disease/model	Evidence
miR-873-3p EVs induced by TSA	HDACi-driven hyperacetylation	Myotubes to BM-MSCs	Promotes osteogenesis and bone mass	Bone regeneration	Preclinical (24)
Exosomal *COL6A1*	H3K27ac-activated *COL6A1*	Osteosarcoma cells to fibroblasts	Induces CAF conversion and metastasis	Osteosarcoma	Tumor model (77)
RAB31-dependent EV release	Promoter H3K9ac	Hepatic stellate cells	Enhances EV release and fibrogenic signaling	Liver fibrosis	Mechanistic (78)
TM4SF5 trafficking	Microtubule acetylation/HDAC6	Cancer cells	Regulates vesicular trafficking and signaling	Cancer migration	Cell model (79)
EV STAT3	STAT3 acetylation/Sec15b	Embryonic stem cells	Modulates STAT3 loading into EVs	Developmental model	Mechanistic (80)
GRP78 secretion	Acetylation-dependent GRP78 release	Colon cancer cells	Alters stress-related cargo secretion	Colon cancer	Cell model (81)
HSPC111	ACLY–acetyl-CoA–H3K27ac axis	CRC cells to CAFs	Induces *CXCL5* and liver metastasis	Colorectal cancer	Mechanistic (84)
FGD5-AS1	p300-mediated STAT3 acetylation	Pancreatic cancer cells to macrophages	Drives M2 polarization and progression	Pancreatic cancer	Immune mech (25).
circ-001422	p300-mediated STAT3 acetylation	Glioma cells to macrophages	Activates STAT3/NF-*κ*B and M2 polarization	Glioma	Immune mech (85).
circ_0006896	HDAC1/H3 acetylation	AML cells/CD8^+^ T cells	Promotes ferroptosis resistance and T-cell suppression	AML	Immune mech (86).
miR-1249	HDAC10/NF-*κ*B–CaSR axis	Endothelial EVs	Regulates pulmonary vascular remodeling	Pulmonary hypertension	Preclinical (87)
Acetylated glycolytic enzymes	Lysine-acetylated metabolic enzymes	Breast cancer small EVs	May affect glycolysis and metabolism	Breast cancer	Acetylome (96)
Acetylated LAP-TGF-*β*1	Acetylated TGF-*β* cargo	TNBC cells	Promotes lung micrometastatic spread	TNBC	Functional (115)

### 4.1 Immunological implications of the EV-acetylation axis in cancer

Because this review is particularly relevant to cancer immunity and immunotherapy, the EV-acetylation axis should also be considered in the context of immune-cell communication and antitumor immune regulation. Tumor-derived EVs can reshape the tumor immune microenvironment by transferring immunosuppressive proteins, nucleic acids, checkpoint molecules and metabolic regulators to immune cells, thereby promoting T-cell dysfunction, immune escape and resistance to immunotherapy (103). Conversely, EVs derived from immune cells may participate in either immune activation or immune suppression depending on their cellular source, activation state and cargo composition. In parallel, protein acylations, including acetylation, have emerged as important regulators of cancer immunity by modulating gene transcription, immune-cell differentiation, inflammatory signaling, cytotoxic effector programs and immune checkpoint pathways (104). These observations indicate that EV-mediated intercellular communication and acetylation-dependent immune regulation are not independent processes, but may converge to shape antigen presentation, macrophage polarization, T-cell exhaustion, regulatory T-cell (Treg) activity, natural killer (NK)-cell dysfunction and response to immunotherapy.

Dendritic cells represent a central interface between EV biology and antitumor immunity because they are professional antigen-presenting cells and key initiators of T-cell responses. EVs can be engineered or naturally loaded with tumor antigens and delivered to dendritic cells to enhance antigen presentation. For example, Dang et al. developed red blood cell-derived EVs targeting splenic DEC-205^+^ dendritic cells and showed that ovalbumin-loaded EVs enhanced MHC class I antigen presentation and antigen-reactive T-cell activation, supporting the potential of EV-based antigen delivery for cancer vaccines (105). In addition, Morisaki et al. reported that neoantigen peptide-pulsed dendritic cell-derived exosomes can induce monocytes to acquire antigen-presenting properties and activate neoantigen-reactive T lymphocytes, suggesting that dendritic cell-derived exosomes may function as endogenous immune amplifiers during neoantigen vaccine therapy (106). Acetylation may further influence this process by regulating dendritic-cell maturation, cytokine production and antigen-presenting capacity. In non-small cell lung cancer, low-dose doxorubicin induced epigenetic remodeling in dendritic cells, including increased H3K14 acetylation, enhanced antigen-presenting ability, IL-12-associated T-cell activation and tumor-protective immune responses (107). However, direct evidence showing that acetylation specifically remodels dendritic cellderived EV cargo to enhance antigen presentation remains limited, and this should be addressed in future studies.

The clearest direct evidence for immune regulation by the EV-acetylation axis currently comes from macrophages and CD8^+^ T cells. As discussed above, pancreatic cancer-derived exosomal FGD5-AS1 interacts with p300 in macrophages, leading to enhanced STAT3 acetylation, activation of the STAT3/NF*κ*B axis, M2 macrophage polarization and tumor progression (25). Similarly, glioma-derived exosomal circ-001422 binds to p300 and promotes STAT3 acetylation, thereby activating the STAT3/NF-*κ*B pathway and driving M2 macrophage polarization (85). These studies provide direct examples of tumor EV cargos reshaping macrophage function through acetylation-related enzymes. In acute myeloid leukemia, EVassociated circ 0006896 binds to the catalytic domain of HDAC1, reduces histone H3 acetylation and alters transcriptional programs linked to ferroptosis resistance. Importantly, the same EV cargo also acts in CD8^+^ T cells, where it impairs *LEF1* transcription and decreases cytotoxic effector molecules such as IFN-*γ* and granzyme B, thereby weakening antitumor immunity (86). Together, these findings demonstrate that tumor EVs can transmit epigenetic regulatory signals to immune cells and directly reprogram acetylation-dependent immune functions.

Beyond macrophages and CD8^+^ T cells, the EV-acetylation axis may also intersect with Treg biology, NKcell cytotoxicity and immune checkpoint regulation, although direct EV-mediated acetylation mechanisms in these cell populations remain less well established. Tumor-derived EVs have been shown to regulate tumor-infiltrating Tregs through the inhibitory immunoreceptor CD300a on dendritic cells, thereby influencing the IFN-*β*-Treg axis in the tumor microenvironment (108). More broadly, EV-mediated crosstalk between cancer cells and T cells can impair effector T-cell immunity while enhancing Treg-associated immunosuppression (109). Since FOXP3 stability and Treg suppressive function are controlled by multiple post-translational modifications, including acetylation, EV-induced changes in Treg activity may potentially intersect with acetylation-dependent FOXP3 regulation (110, 111). NK cells are another important target of tumor-derived EVs. Cancer exosomes can impair NK-cell activation and cytotoxicity by modulating activating receptors, tumor-recognition ligands and immunosuppressive pathways (112, 113). In AML, exosomes containing PD-L1 suppress NK-cell activation and cytotoxicity through the PD-1/PD-L1 pathway, and this effect can be alleviated by PD-L1 blockade (114). EV-mediated immune checkpoint signaling may be further amplified by large EV subtypes, as cancer-derived blebbisomes contain inhibitory checkpoint proteins such as PD-L1, PD-L2, B7-H3, VISTA, PVR and HLA-E (31). Given that acetylation and HDAC activity can regulate immune-cell transcriptional states and checkpoint-associated pathways, these findings suggest that EV-acetylation crosstalk may influence checkpoint blockade responses. Nevertheless, whether tumor EVs directly reshape FOXP3 acetylation, NK-cell acetylation programs or checkpoint-associated chromatin states remains to be directly tested using integrated EV profiling, acetylome analysis and immune functional assays.

### 4.2 Critical appraisal of current evidence and methodological limitations

Although the studies summarized above support the existence of reciprocal regulation between EVs and acetylation, the current evidence should be interpreted with caution. First, many reported mechanisms are derived from individual studies performed in specific tumor types, stromal-cell populations, immune-cell subsets, or non-malignant disease models. For example, H3K9ac-dependent regulation of *RAB31* has been demonstrated in liver fibrosis, whereas exosomal FGD5-AS1- and circ-001422-mediated regulation of STAT3 acetylation has been reported in pancreatic cancer and glioma, respectively (25, 78, 85). These findings provide strong mechanistic examples, but they do not yet establish a universal EV-acetylation program across all diseases or EV subtypes.

Second, EV isolation and characterization methods vary substantially among studies, which may influence the interpretation of EV-associated acetylation signals. Differential ultracentrifugation, precipitation-based methods, size-exclusion chromatography, density gradients and immunoaffinity capture can enrich different EV subpopulations and co-isolate non-vesicular proteins, lipoproteins or ribonucleoprotein complexes.

Therefore, changes attributed to EV cargo should ideally be validated using multiple isolation strategies, EV-depletion controls, protease or RNase protection assays, and functional rescue experiments. In addition, according to MISEV recommendations, EV subtypes should not be assigned solely by size or common markers unless their biogenesis is experimentally supported (30). This issue is particularly relevant when studies refer to “exosomes” while using preparations that may contain heterogeneous small EVs or non-vesicular contaminants.

Third, the strength of evidence differs across different parts of the proposed EV-acetylation axis. Some mechanisms are directly supported by functional experiments, such as EV-mediated delivery of noncoding RNAs that interact with p300 or HDAC1 and alter STAT3 or histone H3 acetylation in recipient immune cells (25, 85, 86). Other mechanisms are plausible but still incompletely validated. For instance, EV-associated acetylated glycolytic enzymes may influence recipient-cell metabolism and lactate-driven epigenetic remodeling, but direct evidence showing that EV-delivered acetylated enzymes induce histone lactylation remains lacking (96). Similarly, although blebbisomes carry immunoregulatory checkpoint proteins, their relationship with acetylation-dependent cargo loading or recipient-cell acetylation remodeling remains unknown (31).

Finally, most available evidence remains preclinical. Few studies have validated EV-associated acetylation markers in large patient cohorts, and even fewer have tested whether therapeutic modulation of acetylation changes clinically relevant EV functions *in vivo*. Future studies should integrate standardized EV isolation, single-vesicle analysis, acetylome profiling, genetic or pharmacological perturbation of acetylation enzymes, and clinically annotated patient samples to distinguish causal mechanisms from correlative associations.

## 5 Biomarker potential of EV-associated acetylation signatures

Beyond their mechanistic roles, EVs may provide minimally invasive biomarkers for monitoring disease activity, immune status and therapeutic response. EVs are present in blood, urine, saliva and other body fluids, and their cargo composition can reflect the physiological or pathological state of donor cells. In cancer, EV-associated proteins, nucleic acids and metabolites have been explored as biomarkers for tumor burden, metastatic potential, immune suppression and treatment resistance (44). In the context of the EV-acetylation axis, several classes of analytes may have biomarker value, including acetylation-related enzymes, non-coding RNAs that regulate acetylation writers or erasers, metabolites linked to acetyl-CoA availability, and acetylated proteins carried by EVs.

Acetylated EV cargo is particularly attractive because it may report both donor-cell signaling and recipient-cell reprogramming potential. For example, breast cancer-derived small EVs contain glycolysisrelated enzymes with distinct lysine acetylation patterns (96), and acetylated LAP-TGF-*β*1 incorporated into exosomes promotes triple-negative breast cancer dissemination (115). These findings suggest that EVassociated acetylated proteins could potentially serve as functional biomarkers rather than passive disease indicators. Similarly, EV cargos that modulate p300, HDAC1, HDAC10, SIRT proteins or acetyl-CoA metabolism may indirectly indicate acetylation-dependent remodeling in the tumor microenvironment (25, 84, 86, 87). In immune-oncology settings, EV-associated checkpoint molecules, immunosuppressive RNAs and acetylation-related cargo may help identify patients with T-cell dysfunction, macrophage polarization or resistance to immune checkpoint blockade.

However, several analytical and translational challenges remain. First, EV biomarker studies are highly sensitive to pre-analytical variables, including sample type, storage conditions, anticoagulants, freeze-thaw cycles, isolation methods and normalization strategies. Second, acetylated proteins may be present at low abundance, and their detection often requires high-quality mass spectrometry, enrichment of acetyllysine peptides, or highly specific antibodies. Third, it remains difficult to determine whether a detected acetylated protein is truly enclosed within EVs, attached to the EV surface, or co-isolated as a non-vesicular contaminant. Therefore, EV-based acetylation biomarkers should be validated using orthogonal approaches, including EV purification, protease protection assays, immunoaffinity capture, single-vesicle analysis and matched tissue or plasma comparisons, in line with current EV characterization recommendations (30).

For clinical translation, future studies should move beyond discovery-stage profiling and establish robust biomarker pipelines. These should include standardized EV collection and isolation protocols, quantitative assays for acetylated cargo, independent validation cohorts, disease-stage stratification and longitudinal sampling before and after therapy. In particular, combining EV cargo profiling with acetylome or metabolomic analysis may help identify biomarker panels that reflect both EV-mediated communication and acetylation-dependent disease states. At present, EV-associated acetylation signatures remain promising but exploratory biomarkers, and their clinical utility will require rigorous validation in prospectively collected patient cohorts.

## 6 Clinical potential of targeting the EV-acetylation axis

The clinical potential of targeting acetylation and its interaction with extracellular vesicles is substantial. Numerous studies have shown that EVs formed under pathological conditions actively contribute to disease progression. Consequently, directly targeting and inhibiting EVs has emerged as a viable therapeutic strategy. In general, such strategies can be divided into three categories: (1) eliminating circulating EVs, (2) inhibiting their secretion, and (3) preventing their uptake by recipient cells.

### 6.1 Inhibition of pathological EV production, circulation and uptake

One promising approach involves removing cancer cell-derived EVs from the circulation. Studies have demonstrated that targeting surface molecules present on EVs, such as HER2, CD9, and CD63, can effectively deplete tumor-derived EVs and thereby inhibit metastasis (116, 117). In parallel, several investigations have focused on inhibiting EV biogenesis and secretion as an anticancer strategy. For example, the neutral sphingomyelinase inhibitor GW4869 and other nSMase2 inhibitors have shown potential in reducing EV secretion, which, in turn, can limit cancer cell metastasis (118).

In addition to targeting EV release, blocking EV uptake offers another therapeutic avenue. The internalization of EVs is facilitated, at least in part, by surface molecules such as heparan sulfate proteoglycans (HSPGs) (120). Inhibiting these receptors or associated endocytic pathways could potentially prevent the spread of cancer-promoting signals. However, EV recognition and uptake remain major unresolved bottlenecks for clinical translation, particularly for engineered EV therapies. The mechanisms that determine recipient-cell selectivity, surface binding, internalization, intracellular trafficking, and productive cargo release are still incompletely understood. This uncertainty is especially relevant to engineered EVs designed to deliver acetylation-modifying RNAs, proteins, small molecules, or epigenetic regulators, because therapeutic efficacy depends not only on EV accumulation in target tissues but also on functional cargo delivery to the appropriate recipient cells. Therefore, future studies should define the molecular determinants of EV tropism and uptake and establish functional readouts that directly link EV delivery to changes in acetylation states in recipient cells.

Traditional Chinese medicine and natural compounds, owing to their complex chemical compositions and multi-target actions, also present innovative opportunities for modulating EVs in modern medical practice. Recent research has highlighted that certain natural compounds, such as berberine, can inhibit EV generation and release, thereby helping to slow the progression of various diseases (121). Although the detailed links between these agents, EV biogenesis, and acetylation remain to be fully elucidated, they provide a promising resource for discovering new modulators of the EV-acetylation axis.

### 6.2 Therapeutic and regenerative EVs for restoring acetylation homeostasis

While EVs produced under pathological conditions usually accelerate disease progression, EVs derived from specific beneficial cell types or generated under defined therapeutic conditions may ameliorate disease phenotypes. For example, newt cell-derived EVs and other regenerative EVs have been explored as cardioprotective agents, highlighting the potential to harness beneficial EVs for tissue protection and repair (119). In addition, EVs derived from mesenchymal stem cells, endothelial progenitor cells, or other reparative cell populations may exert therapeutic effects by modulating inflammatory, metabolic, and epigenetic programs in recipient cells.

Emerging evidence suggests that the therapeutic effects of these EVs are often associated with the modulation of acetylation. For example, Huang et al. reported that EVs secreted by bone marrow endothelial progenitor cells can alleviate diabetes-induced vascular dysfunction. In diabetic conditions, cardiac endothelial cells exhibit transcriptional repression of genes involved in angiogenesis, proliferation, and survival, partly due to HDAC-mediated reduction of H3K9 acetylation. This repression impairs the reparative function of endothelial progenitor cell-derived EVs in ischemic heart tissue. Their results indicate that EVs can help prevent diabetes-induced vascular dysfunction by normalizing intracellular acetylation processes (122). Similarly, HDAC inhibition has been shown to alter the functional output of EVs. For instance, TSA-induced hyperacetylation promotes the secretion of miR-873-3p-enriched EVs that enhance osteogenic differentiation (24). These findings support the concept that therapeutic EVs may be used to restore acetylation homeostasis in diseased tissues.

### 6.3 HDAC inhibitors, EV-based therapeutics and combination strategies

Considerable progress has been made in exploiting acetylation modifications, particularly through HDAC inhibition, for disease treatment. Several HDAC inhibitors have entered clinical practice, mainly in hematological malignancies. Examples include vorinostat and romidepsin for cutaneous T-cell lymphoma, belinostat for peripheral T-cell lymphoma, and panobinostat for multiple myeloma, whereas other HDAC inhibitors are being evaluated in ongoing or completed clinical trials for solid tumors and combination regimens (72, 124). These agents increase acetylation levels of histone and non-histone proteins and thereby influence transcription, cell-cycle progression, apoptosis, DNA damage responses and antitumor immunity (123). However, the clinical activity of HDACi remains context dependent. Their broader application is limited by incomplete isoform selectivity, dose-limiting toxicities, hematological and gastrointestinal adverse effects, acquired resistance, compensatory epigenetic pathways and heterogeneous responses across tumor types (72, 124).

EV-based therapeutic approaches are also advancing, but most remain at the preclinical or early clinical stage. Potential strategies include inhibiting pathological EV production or uptake, removing circulating tumor-derived EVs, using naturally therapeutic EVs from reparative cells, and engineering EVs as delivery vehicles for RNAs, proteins, small molecules or epigenetic regulators (27, 28). Compared with synthetic nanoparticles, EVs may offer advantages such as intrinsic biocompatibility, membrane-based targeting properties and the ability to transfer complex biological cargo. Nevertheless, major translational barriers remain, including scalable production, batch-to-batch reproducibility, cargo loading efficiency, EV heterogeneity, biodistribution, storage stability, potency assays, safety evaluation and regulatory standardization.

Given the mechanistic relationship between EVs and acetylation, combining HDACi or other acetylation modulators with EV-targeted interventions may offer rational therapeutic opportunities. For example, inhibition of pathological EV release or uptake may reduce the dissemination of tumor-promoting and immunosuppressive signals, whereas HDACi may simultaneously remodel acetylation-dependent transcriptional programs in tumor, stromal and immune cells. Conversely, engineered EVs designed to modulate acetylation, including those delivering SIRT1-targeting cargo or other epigenetic regulators, have shown promising therapeutic effects in animal models (125). However, combined HDACi and EVtargeting strategies have not yet been clinically validated as a unified therapeutic modality. Future studies should define whether HDACi alter EV secretion, cargo composition, immunogenicity or biodistribution in patients, and whether EV-based delivery can improve the therapeutic index of acetylation-targeting agents.

These therapeutic modalities include pathological EV inhibition, regenerative EV administration, engineered EV delivery and pharmacological modulation of acetylation. Rather than being mutually exclusive, these approaches may represent complementary strategies for targeting the EV-acetylation axis. A rational clinical strategy may ultimately involve their sequential or combinatorial deployment depending on disease stage, tissue context, EV source, immune status and the specific acetylation abnormalities driving pathology.

## 7 Conclusions and future perspectives

Accumulating evidence indicates that extracellular vesicles and acetylation are tightly intertwined regulators of disease progression. As central mediators of intercellular communication, EVs participate in diverse pathological processes, including cancer, neurodegenerative disorders, and cardiovascular disease. In parallel, acetylation serves as a dynamic post-translational and epigenetic regulatory layer that shapes chromatin architecture, transcriptional programs, and non-histone protein function. Recent studies have revealed that acetylation can control EV biogenesis, cargo sorting, secretion, and EV-associated protein trafficking (24, 77, 78, 80), while EVs can reprogram acetyl-CoA metabolism and acetylation machinery in recipient cells through the transfer of RNAs, proteins, metabolites, and acetylated proteins (25, 84, 86, 96, 115). This bidirectional EV-acetylation axis provides a unifying framework for understanding how local and systemic microenvironments are remodelled during disease and may help explain the context-dependent effects of HDACi that have already entered clinical use.

Looking ahead, several lines of investigation will be critical to translate these insights into therapeutic benefit. First, a more detailed mechanistic dissection of how specific EV cargos, such as non-coding RNAs, acetylation-related enzymes and acetylated proteins, shape acetylation landscapes in defined cell types and disease stages is required to identify tractable nodes within the EV-acetylation network. This is particularly important in tumor immunity, where EV-acetylation crosstalk may influence antigen presentation, macrophage polarization, T-cell dysfunction, Treg activity, NK-cell cytotoxicity and immune checkpoint signaling. Second, EV-associated acetylation signatures should be evaluated as minimally invasive biomarkers for patient stratification and treatment monitoring, but this will require standardized EV isolation, rigorous acetylome profiling, independent validation cohorts and longitudinal clinical sampling. Third, rationally designed strategies that combine HDACi or other epigenetic modulators with EV-targeted interventions may help overcome the limited selectivity, toxicity and resistance that currently restrict HDACi monotherapy. These interventions may include inhibition of pathological EV release or uptake and the use of engineered EVs as delivery vehicles for acetylation-modifying cargos. At the same time, the field must distinguish experimentally validated mechanisms from model-specific observations and speculative links before EV-associated acetylation biomarkers or combined EV-HDACi strategies can be translated into clinical practice. Although substantial challenges remain, including EV heterogeneity, inter-patient variability and the need for robust clinical validation, targeted manipulation of the EV-acetylation axis is poised to become an important component of future precision therapies across a broad spectrum of diseases.
